# A Process for Digitizing and Simulating Biologically Realistic Oligocellular Networks Demonstrated for the Neuro-Glio-Vascular Ensemble

**DOI:** 10.3389/fnins.2018.00664

**Published:** 2018-09-25

**Authors:** Jay S. Coggan, Corrado Calì, Daniel Keller, Marco Agus, Daniya Boges, Marwan Abdellah, Kalpana Kare, Heikki Lehväslaiho, Stefan Eilemann, Renaud Blaise Jolivet, Markus Hadwiger, Henry Markram, Felix Schürmann, Pierre J. Magistretti

**Affiliations:** ^1^Blue Brain Project, École Polytechnique Fédérale de Lausanne (EPFL), Geneva, Switzerland; ^2^Biological and Environmental Sciences and Engineering Division, King Abdullah University of Science and Technology, Thuwal, Saudi Arabia; ^3^Visual Computing Center, King Abdullah University of Science and Technology, Thuwal, Saudi Arabia; ^4^CRS4, Center of Research and Advanced Studies in Sardinia, Visual Computing, Pula, Italy; ^5^CSC – IT Center for Science, Espoo, Finland; ^6^Département de Physique Nucléaire et Corpusculaire, University of Geneva, Geneva, Switzerland; ^7^The European Organization for Nuclear Research, Geneva, Switzerland

**Keywords:** electron microscopy, 3D reconstruction, simulation, NGV, energy metabolism, *in silico* visualization

## Abstract

One will not understand the brain without an integrated exploration of structure and function, these attributes being two sides of the same coin: together they form the currency of biological computation. Accordingly, biologically realistic models require the re-creation of the architecture of the cellular components in which biochemical reactions are contained. We describe here a process of reconstructing a functional oligocellular assembly that is responsible for energy supply management in the brain and creating a computational model of the associated biochemical and biophysical processes. The reactions that underwrite thought are both constrained by and take advantage of brain morphologies pertaining to neurons, astrocytes and the blood vessels that deliver oxygen, glucose and other nutrients. Each component of this neuro-glio-vasculature ensemble (NGV) carries-out delegated tasks, as the dynamics of this system provide for each cell-type its own energy requirements while including mechanisms that allow cooperative energy transfers. Our process for recreating the ultrastructure of cellular components and modeling the reactions that describe energy flow uses an amalgam of state-of the-art techniques, including digital reconstructions of electron micrographs, advanced data analysis tools, computational simulations and *in silico* visualization software. While we demonstrate this process with the NGV, it is equally well adapted to any cellular system for integrating multimodal cellular data in a coherent framework.

## Introduction

### Overview

We present a snapshot of progress in the development of a universal process for the creation of morphologically accurate digital reconstructions of a functional assembly of cells for the purpose of conducting biologically realistic computer simulations and *in silico* experiments. Our goal is to explore and understand cooperative biochemical and biophysical functions of networks of several cells (oligocellular networks). Specifically, we focus on the re-creation of the neuro-glio-vasculature (NGV) ensemble and our multi-scale energetics modeling program. Although we apply this system to our particular interest in brain energy metabolism, our procedure can be applied to any oligocellular grouping from any tissue or species. This innovative procedure uses state-of-the-art techniques and software developed primarily by a collaborative alliance between the Blue Brain Project (BBP) of the École Polytechnique Fédérale de Lausanne (EPFL), in Switzerland, and the King Abdullah University of Science and Technology (KAUST), in Saudi Arabia, with the aim of facilitating the advancement neuroscience with *in silico* methods. The workflow, designed in 4 stages, addresses previous inadequacies in resolution and accuracy of imaging, seeks to automate many labor-intensive steps, implements multi-scale modeling and uses advances in *in silico* imaging and large-scale visualization techniques, including virtual reality (VR), as research tools.

### Cytoscale *in silico* Neuroscience

Simulation-based research, often referred to as the *in silico* method, is establishing itself as an indispensable tool to bridge physical and temporal scales, enhance insights and accelerate progress in more and more scientific disciplines. In biology, it has long been recognized that mathematical modeling would be required to complement experimental efforts to understand basic principles of life’s complexity, ranging from cellular phenomena (Hodgkin and Huxley, 1952; Del Castillo and Katz, 1954; Joyner et al., 1978; Bartol et al., 1991; Stephanova and Bostock, 1995; Tsodyks and Markram, 1997; Nadkarni and Jung, 2007; Coggan et al., 2010; Xylouris and Wittum, 2015) to network behavior (e.g., Eliasmith et al., 2002; Izhikevich, 2004; Migliore et al., 2006), just to name a few. This realization has engendered the creation of multiple simulation environments to address this need (Bartol et al., 1991; Hines and Carnevale, 1997; Bower and Beeman, 1998; Tomita et al., 1999; Schaff et al., 2000; Eppler et al., 2008). Our simulation niche, involving a few interacting cells, is often given the cytoscale designation, an intermediate level between molecular and higher-order network models.

There are many approaches to modeling in biology depending on the question, goal and constraints (Brodland, 2015). For instance, cellular shapes and spatial organization are critical to normal physiology. Ultrastructural characteristics may impinge upon computations and other functions from the single cell to the network level. Cells cannot, therefore, be understood within the boundaries of a box-like single compartment model with well-mixed biochemical reactions. The limitations of geometrically and spatially simplistic models entreats a better approach: thinking “outside-the-box,” so to speak, requires thinking about what’s happening “inside-the-box”; one must consider the complexity of the internal cellular complement of organelles and macromolecular complexes, as well as local “virtual” micro-environments created by, for example, restricted diffusion, lipid-water interfaces and non-uniform protein expression (e.g., Berridge, 2006; Shillcock, 2008).

Perhaps the most prevalent method for discovering the micro-architecture of life has been electron microscopy (EM). This technique has a storied past in the annals of cell biology from the first pictures of eukaryotic cells (Porter et al., 1945) to the pioneering studies on organelles such as mitochondria (Palade, 1952), discernment of macromolecular processes such as muscle contraction filaments (Huxley, 1957), and proof for the neuron doctrine (Palade and Porter, 1954). Of great importance to neuroscience have been the demonstration of pre-synaptic vesicles (De Robertis and Bennett, 1955), the first entire nervous system (White et al., 1986) and the possible structural correlates of learning (Trachtenberg et al., 2002; de Vivo et al., 2017). No less important to neuroscience are the astrocytes (Vaughn and Pease, 1967; Bushong et al., 2004) and the particulars of their subcellular milieu such as the localization of the glycogen macromolecular complexes that are so critical to the energy supply to brain tissue (Oe et al., 2016) – this latter type of data requiring advanced three-dimensional (3D) EM techniques (Calì et al., 2016, 2017; Calì, 2017). The importance of the 3D arrangement of astrocytic processes has been evident since the 1960s when early observations of astrocytes from serial section EM appeared (Wolff, 1965; Stensaas and Stensaas, 1967; Poritsky, 1969).

By taking the detailed 3D environment of biological systems into consideration in addition to kinetic properties, a spatial-temporal simulation approach becomes superior for revealing and understanding fundamental principles. The efficacy of this synergism has been demonstrated at various scales. Fundamental functions that could not have been measured, observed or predicted based on experiments alone have emerged from this kind of simulation from the sub-cellular level toward one end of the domain scale (e.g., Coggan et al., 2005; Keller et al., 2008; Bartol et al., 2015), to the mammalian cortical circuit level toward the other end (e.g., Markram et al., 2015; Reimann et al., 2017), while the most comprehensive effort to create an anatomically and physiologically realistic simulation of an entire organism, demonstrated in the roundworm *Caenorhabditis elegans* by the OpenWorm project^1^, has witnessed the first re-creations of animal behaviors with a predictive modeling approach (Szigeti et al., 2014). In these and other cases, both anatomical and physiological accuracy were of paramount importance for the bottom-up elaboration of complex behaviors to eventually achieve the aim of life-like simulations. As the late, illustrious physicist Richard Feynman keenly pointed-out: “What I cannot create, I do not understand.”

### Application to Brain Energy Metabolism

Our scientific goal is to establish an integrated workflow of advanced tools and procedures to facilitate the investigation of the role of cytoscale structures and functions in the management of brain energy metabolism, thereby advancing the speed and accuracy of this critical aspect of neuroscience research. We describe the current state and application of our anatomical capture and simulation processes to create a software infrastructure that allows the use of supercomputers as interactive scientific instruments for wide adoption by scientists. We then use this technology to advance our detailed knowledge of the coupling between the cellular elements of the NGV and use this knowledge for future energy-efficient computing paradigms (e.g., Conrad et al., 2018).

Despite the importance of energy supply to normal brain function and its dysfunction in disease (Harris et al., 2012; Harris J.J. et al., 2015), much remains unknown about how neurons and astrocytes share resources or what the mechanisms of cerebral blood flow (CBF) regulation by brain activity are. The NGV ensemble acts as a functional unit with each component carrying-out delegated tasks as circumstances dictate. The energy dynamics of the brain require the direct supply of all cells with energy as well as a system that allows cell-to-cell energy transfers as needed. The modeling of brain energy metabolism is one of the best subjects in which a dual structure-function approach should be applied as it (i) is a universal requirement of all brain functions, (ii) is dependent on the cooperative engagement of several cell types, and (iii) stands as a measure of local brain activity (Buxton, 2010; Hillman, 2014; Hyder and Rothman, 2017).

While the primary food for the brain is simply glucose, what happens after the passage of that energy-rich molecule from the vasculature into the brain is far from a simple matter (Pellerin and Magistretti, 2012). In addition, glucose can be anaplerotically injected into the energy stream on demand through glycogenolysis, the breakdown of the glycogen granules stored primarily in astrocytes into glucosyl subunits (Coggan et al., 2018). Although glycogen is used primarily as a glucose reservoir in peripheral organs, there is mounting evidence for more specialized and dynamic functions in the brain (Gruetter, 2003; Walls et al., 2009; Waitt et al., 2017). As in most cells, glucose and its metabolites pinball through a series of reaction steps that extract chemical energy through reducing equivalents to support the brain’s needs. But apparently unique to the brain is an intricate and coordinated division of labor allocated by cell type. These labor laws are written in the differential expression and regulation of the enzymes and transporters that comprise the metabolic gauntlet (e.g., Pellerin and Magistretti, 1994; Bittar et al., 1996; Dringen et al., 2000; Pierre and Pellerin, 2005; Magistretti and Allaman, 2007; Herrero-Mendez et al., 2009). Disobeying these rules is detrimental to proper function, for example, in the case when glycogen is over-expressed in neurons in Lafora disease, a deadly form of juvenile epilepsy (Vilchez et al., 2007). Anomalies in brain metabolism are observed in almost all neuropsychiatric disorders, in particular those that are neurodegenerative (Harris et al., 2012; Stobart and Anderson, 2013; Barros et al., 2017).

Based on the many key roles of astrocytes in sensing neuronal activity and its coupling to energy delivery, the capability to model astrocyte energy usage, production and delivery would enable better and broader constraints for many simulations including cortical neuronal models (Magistretti, 1988; Pellerin and Magistretti, 1994; Magistretti and Pellerin, 1996, 1999; Hillman, 2014; Markram et al., 2015). With the current NGV model, we can identify the contributions of each cell-type to brain energy usage and management. In neurons, glucose enters glycolysis as it would in any cell, but midway through byproducts are shunted away and denied further processing in the oxidative tricarboxylic acid cycle (TCA) (Bolaños and Almeida, 2010). The astrocyte’s metabolic profile, in contrast, favors the production of lactate from pyruvate (Walz and Mukerji, 1988; Bélanger et al., 2011). The lactate can then be exported to a neighboring neuron in a process called the astrocyte-to-neuron-lactate shuttle (ANLS; Pellerin and Magistretti, 1994, 2012; Magistretti and Pellerin, 1996; Brooks, 2009; Jolivet et al., 2009, 2015) where it is converted back to pyruvate for fueling the TCA cycle in the mitochondria of the neuron. The transfer of lactate to neurons is critical for the normal function of the brain including memory and learning (Suzuki et al., 2011; Wyss et al., 2011; Boury-Jamot et al., 2016a; Gao et al., 2016; Steinman et al., 2016), stabilization of mood (Carrard et al., 2016) and proper sleep (Baud et al., 2016), whereas the exogenous application of lactate or its precursor pyruvate may reduce the severity of traumatic brain damage (Glenn et al., 2015; Shijo et al., 2017).

To achieve our aim, we must use a battery of techniques in multiple steps to re-create neurons, astrocytes (and ultimately other glial cells, too) and the microvasculature as the stage for molecular simulations in which we deploy high-precision, multi-scale *in silico* methods within the morphologically realistic representations of this cellular energy management cartel. In addition, we incorporate *in silico* visualization of the data in all its forms to gain a quantitative understanding of function that will inform future research.

## Process Description

We break our biologically realistic 3D modeling process down into a 4-stage workflow framework (WF) that includes EM data acquisition (WF1) that lays the foundation for the subsequent stages by providing the essential morphological data required for reconstruction of the NGV microenvironment. In WF2, structural and comparative analysis, we utilize WF1 morphologies for analysis and development of new analytical tools. Next, we proceed to multi-scale NGV modeling within stereotyped geometrical compartments (WF3), as a prelude to our eventual goal of ultrastructurally realistic delimited simulations. And finally, WF4 is the *in silico* imaging and visualization in which we convert meshes of the morphologies and simulation output into images and VR experiences.

### WF1 – Electron Microscopy and 3D Reconstruction

The work on astrocyte imaging, reconstruction and analysis was the first stage of our framework. The generation of high-resolution models of glial cells is the first, fundamental step toward being able to create an appropriate “scaffold” for *in silico* experiments and voxelize 3D space to compartmentalize metabolic simulations. The spatial complexity of these cells pointed us to the use of EM for imaging, as this technique is the only one providing micrometer (μm)-scale resolution of cellular details. Because of the size and the complexity of the datasets, image processing and visualization from this workflow stage presented challenges that are answered with new technical solutions, as described below in WF2 and WF4 sections.

**FIGURE 1:**
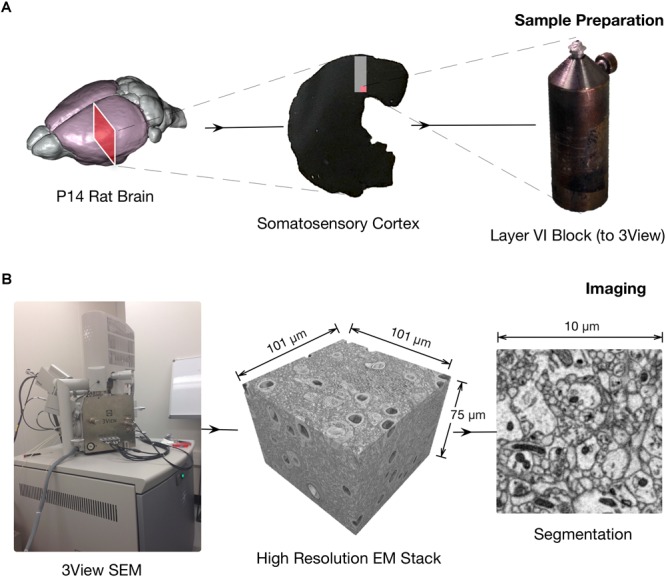
Schematics of the 3DEM pipeline. **(A)** Sample preparation. Rodents brains are processed following cardiac perfusion (left) and then 100 μm thick coronal section containing somatosensory cortex are prepared for face-block scanning EM (center). The section is then placed on a metal stub and trimmed to expose a squared surface of roughly 0.3 to 0.5 millimeters (right). **(B)** Images are obtained using a serial-block face electron microscope, such as FIBSEM or a Quanta 600 SEM equipped with a Gatan 3View module (pictured on the left). Hundreds to thousands serial section micrographs, with a relatively high field of view if necessary can be obtained automatically in a few days with minimal human supervision (center) at high resolution (right).

The main steps of this workflow stage (**Figure 1**) that we will describe in detail are:

• Acquisition of serial electron microscopy images
  ◦ Focused ion beam – scanning electron microscopy (FIB-SEM)
  ◦ 3View - Serial Block-Face Electron Microscopy (3View)

• Image stack processing
  ◦ Stitching
  ◦ Registration

Astrocytes have profound influence over neuronal function and synaptic activity (Magistretti and Ransom, 2002). In order to model and simulate their function in space and time, astrocyte morphologies can be extracted from serial EM image stacks. In the last decade, many automated serial EM techniques have been developed, driven by the need of connectomics to both detect single synaptic contacts (high resolution EM) and also image large portions of the brain of different species (ideally, the eventual goal being to acquire an entire human brain, Knott and Genoud, 2013). In simple words, state-of-the-art EM setups can automatically cut serial sections and image them to produce aligned stacks, with minimal human supervision. Due to technical limitations of the different techniques, we first defined our needs, and then selected the most appropriate tool to do the automated sectioning and imaging of the available brain samples.

**FIGURE 2:**
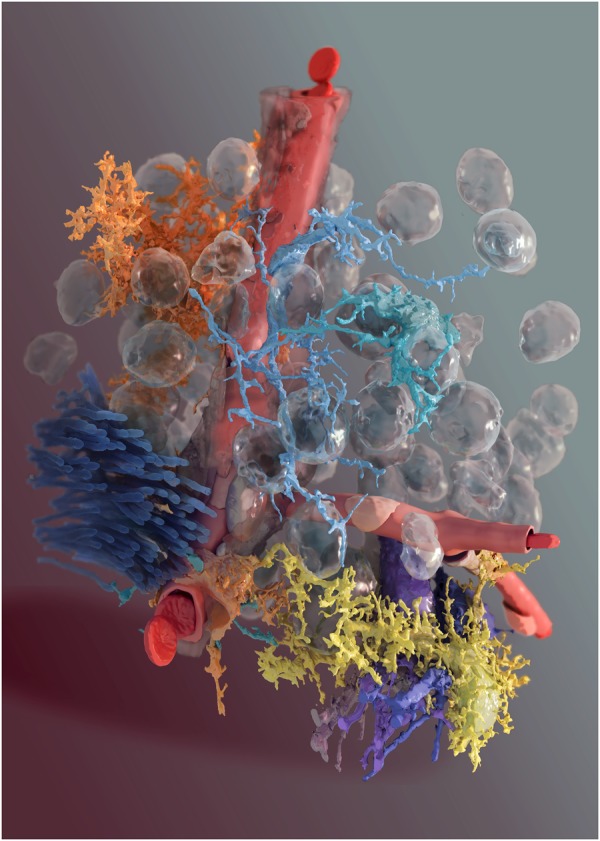
3D reconstruction of glial cells and neurons from Layer VI somatosensory cortex brain parenchyma. A digital reconstruction of a micrograph stack from Layer VI of the somatosensory cortex of a P14 rat. Blood vessel in red, two neurons in shades of violet, two microglia in light blue/green, the fiber tract in dark blue, the pericyte in orange at the bottom of the image and two astrocytes, one in yellow and another one in orange, at the top of the image. Glass-like spheres are the nuclei reconstructed from the original image stack.

**FIGURE 3:**
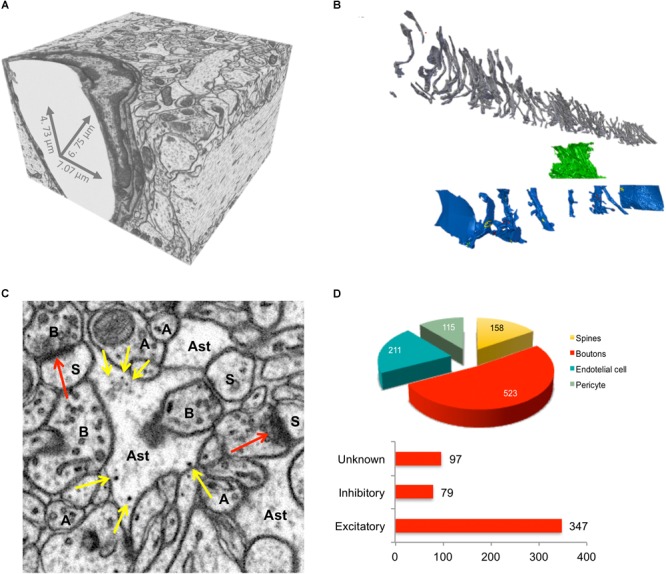
Imaging and quantification of glycogen distribution in EM. **(A)** Isotropic EM stack volume from CA1 rat hippocampus from Calì et al., 2016. **(B)** Exploded view of dense reconstruction from **(A)** Gray, 161 axons; Green, astrocytic process; Blue, 11 dendrites. **(C)** Magnification of a single micrograph from **(A)** showing elements of interest in the neuropil of rat hippocampal CA1 region. Synapses (red arrows), glycogen granules (yellow arrows), axons (A), boutons (B), dendrites (D), spines (s) and astrocytic processes (Ast) on EM micrographs. **(D)** Quantification of the spatial distribution of glycogen granules from sample C; top, number of glycogen granules associated to elements of the neuropil, calculated using a nearest neighbor rationale. Bottom, total number of glycogen granules per bouton type.

We needed to describe both the relationship between the morphologies of entire astrocytes and their environment (**Figure 2**), as well as between astrocytic processes and synapses (**Figure 3**). Our choice was to base the image acquisition on serial block-face EM (SBEM; scheme in **Figure 1**), a technique using a scanning electron microscope (SEM) to detect back-scattered signal from the block-face of a sample to obtain images similar to the more familiar and traditional transmission EM (TEM) images (Deerinck et al., 2018). In order to produce serial sections, we took advantage of two setups: either a focused ion beam (FIB) or a 3View module. The first, called FIB-SEM system, allows for high-resolution imaging and an isotropic (or nearly) voxel size, because the ion beam can cut as thin as 5 nanometers (nm) in optimal conditions. On the other hand, the boundary artifacts showing if the region is larger than 15–20 μm limit its field of view because the cut region is focused on a small region of the block. This makes it unfit for large regions of interest. The latter setup, called 3View, uses a diamond knife placed inside the imaging chamber that physically cuts the whole surface of the sample (**Figure 1B**). Technical limitations like the electrical charge of the sample causing heating and possibly softening or melting of the resin can affect the minimum thickness of the sample, which can hardly be thinner than 20 nm, but such a technique is fit for imaging very large, or multiple regions of interests.

An EM image stack obtained from somatosensory cortex samples from P14 rats using the 3View SEM (**Figure 1**) enabled the reconstruction of the entire 3D morphology of glia and neurons, at an unprecedented level of detail (**Figure 2**). This was possible because the 3View SEM allowed the acquisition of a large field-of-view (100 × 100 × 75 μm), enough to contain entire cells. The use of electron micrographs also allows visualization of the finest, lamelliform processes that are not resolvable with light microscopy. These structures are of particular importance because they interface and modulate synapses. The size and complexity of image data prevent an easy, on-line use of automated or semi-automated segmentation tools, proving an immediate feedback (within seconds) of the segmented structures; at the same time, manual segmentation is tedious and time consuming. For this purpose, we engineered a hybrid pipeline involving a first, rough segmentation that would run offline, followed by a manual proofreading phase (Holst et al., 2016). This will be described in the following section.

From a dataset published in a previous work (Calì et al., 2016), we were able to reconstruct every single axon, dendrite, and astrocytic process included in a 220 μm^3^ portion of neuropil of adult rat hippocampus (**Figure 3**), using the semi-automatic software ilastik (Sommer et al., 2011). This model allowed us to quantify intracellular features and make speculations down to the molecular level, which is essential for modeling the brain (Calì et al., 2016). We are creating tools that are able to interactively measure, cluster, and analyze subcellular features within Blender, a free, open source 3D modeling software package. These tools have been applied to many tasks including analyzing the distribution of the energy-storing glycogen granules within astrocytes (**Figure 3C**). Our analysis revealed that glycogen granules are preferentially polarized toward axonal boutons (**Figure 3D**), suggesting that pre-synaptic terminals are either more energetically expensive than dendritic spines or inaccessible to localized mitochondria. Work is now underway to collect more glycogen granule data from duplicate samples to judge the generality of this observation. As the amount of 3D mesh cell information has increased, the need to efficiently annotate biological features on them has become more pressing. Additional information required includes biological meta-information about reconstructed cells, their components, relationships to each other, and whether they are complete or cut by the edge of the EM stack.

### WF2 – Segmentation and Analysis

Following EM Imaging, as described in the previous section (WF1), we need to proceed with image segmentation, meshing (i.e., 3D reconstruction) and 3D analysis of the morphologies to prepare the synthetic *in silico* version of the cells. The main steps of this workflow are:

• Segmentation and 3D reconstruction
  ◦ Semi-automatic (ilastik)
  ◦ Manual (TrakEM)
  ◦ Hybrid, rough semi-automated and manual proofread
• Visual qualitative analysis tools
• Customized Quantitative analysis and tool creation in Blender

3DEM provides rich and complex information that is challenging for analysis and visualization (Neuro Cloud Consortium, 2016). The main goal of this phase of the process is to provide neuroscientists with the necessary framework to study fine brain cell (neurons and glia) morphology at very high resolution and in 3D volumes in an interactive and intuitive manner in order to extract relevant statistics about the morphologies and generate accurate *in silico* cellular representations.

#### Segmentation and 3D Reconstruction

**FIGURE 4:**
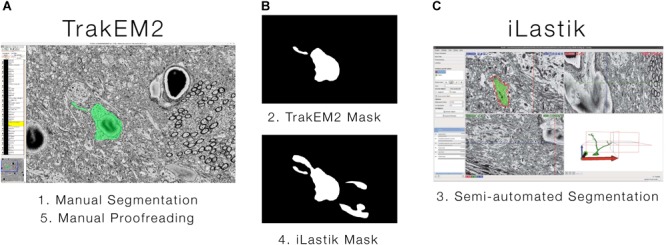
Hybrid semi-automated large-scale dense reconstruction. Our pipeline combines and extends two public domain software programs for segmentation. **(A)** TrakEM2 is used for manually specifying exact boundaries and fine details. **(B)** A binary mask (top) can be extracted from TrakEM2 after a first, detailed segmentation of a structure of interest (a neuron in the example in a, green). **(C)** Ilastik will use the TrakEM2 binary mask as training for a rough but extensive segmentation of most part of the cell along the *z*-axis. The segmentation can be then exported again as binary mask (**B**, bottom), to be imported again in TrakEM2 for further proofreading. The sequence of steps is indicated below each panel and numbered from 1 to 5.

Ultrastructural 3D datasets covering distances of tens or even hundreds of μm are becoming readily available in many laboratories (Helmstaedter et al., 2013; Tomassy et al., 2014; Kasthuri et al., 2015; Harris K.M. et al., 2015; Wanner et al., 2016). However, as datasets have grown in size, the bottleneck for most studies has moved to image processing and analysis (Borrett and Hughes, 2016). In this context, largescale automatic methods for segmentation and 3D reconstruction are becoming available, but as for now, the most common processing pipelines used by many labs to date are *de facto* semi-automated, and still require extensive manual, time-consuming proof-reading efforts (Knowles-Barley et al., 2011; Haehn et al., 2014; Kaynig et al., 2015). Hence, significant resources are invested to improve the state-of-the-art of EM data segmentation, in terms of accuracy, computation time and human effort. Our personal choice was to rely on currently available semi-automatic solutions, such as ilastik (Sommer et al., 2011) whose goal is to reach a segmentation accuracy comparable to what is obtainable with manual tools (TrakEM2; Cardona et al., 2012). Toward this aim, the existing ilastik image classification and segmentation tool was enhanced to cope with the actual needs of the user community. Ilastik^2^ is an open-source, semi-automated image processing tool that is under constant, active development and is a target for on-going image processing research; its architecture and UI are well-suited for ultrastructural 3D datasets. Unfortunately, ilastik’s carving workflow is not scalable with large image stacks. As an alternative solution, TrakEM2, fully manual software for cell segmentation, can be used, but it is very labor intensive and orders of magnitude slower. The pipeline we designed combines the complementary strengths of ilastik and TrakEM2 (Holst et al., 2016). ilastik is good for quickly finding the gross features and processes of a cell, while TrakEM2 is good for specifying exact boundaries and finer details (**Figure 4A**). With little training, ilastik is able to produce an acceptable segmentation for most of the cell (**Figure 4C**); however, each refinement can remove some of the satisfactory areas, requiring additional training (a kind of “two-steps forward, one step back” routine; see numbered steps in **Figure 4**). Combining the tools provides the benefits of both and enables segmentation to proceed in a way that is monotonically improving. As main customization, a novel *mask import* feature was developed in ilastik, which enables a workflow incorporating both tools. Briefly, a segmentation on one or few sections from the cell of interest (i.e., the “mask,” **Figure 4B**) can be done manually in TrakEM2, and then imported into ilastik, that would use it as training to segment the entire cell. Likewise, the segmentation can be exported as a mask from ilastik, then imported into TrakEM2 for further proofreading A novel solution was also designed for dealing with large datasets on a single machine by subdividing them in piecewise chunks to fit with the ilastik semi-automated segmentation module called *“carving,”* which was accordingly refactored (Holst et al., 2016).

**FIGURE 5:**
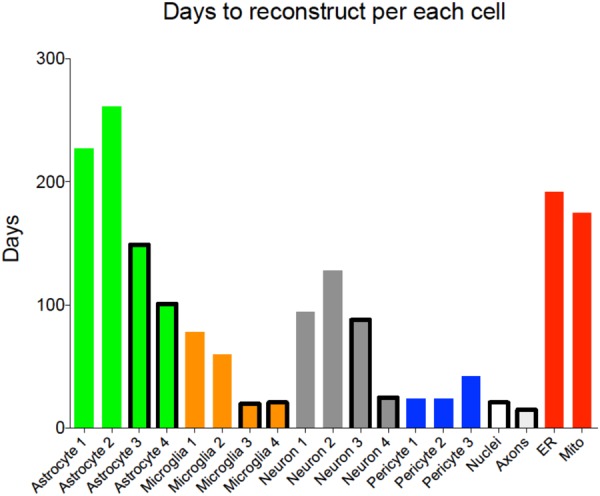
Reconstruction time per each cell type. Histogram showing time to complete each reconstruction, including training of naïve reconstructors and proofreading. Outlined bars highlight cells reconstructed using the new pipeline using ilastik as a first pass segmentation.

The proposed block-wise, out-of-core approach was successfully tested to segment cells in the large KB-E0010 EM stack (4096 × 4096 × 1528 pixels) on a large memory machine (512GB). The main achievements were a substantial reduction of memory usage and a drastic reduction of segmentation times (**Figure 5**). The proposed automated framework eases the timely segmentation of EM stacks and the even larger datasets they will represent. Beyond advanced edge-detection, the solution also supports a manual refinement workflow, allowing segmentation intervention that only a trained and expert neurobiologist can provide.

#### Visual Qualitative Analysis Tools

Interactive explorations, comparisons and queries on cellular structures and complex datasets can be easily achieved using dedicated visualization tools. These needs resulted in the development of tool sets and methods to query, view and explore our EM Stacks, 3D reconstructions, and glial morphology data (Beyer et al., 2013). Tools such as NeuroLines (Al-Awami et al., 2014) or ConnectomeExplorer (Beyer et al., 2013) currently provide the basis for neural connectomics research and enable visual queries over massive datasets of neurite connections, volumetric visualization of petabyte-sized images, and statistical summaries. In the context of this framework, the domain-specific parts of these tools were further extended to support glial cell connectivity (e.g., adding pericytes and vasculature structures) in order to boost the required explorative analysis, and also to improve data validation and productivity. For structures like astrocytes, particularly complex from a geometrical and topological point of view, novel methods for extracting connectivity information were considered (Tagliasacchi et al., 2016), as well as fractal dimensions and porous structure representations (Aboulhassan et al., 2015). For high-resolution volume datasets, multiscale representations were used in order to enable real time interactive exploration on commodity platforms and scale tools depending on the data size (Hadwiger et al., 2018).

**FIGURE 6:**
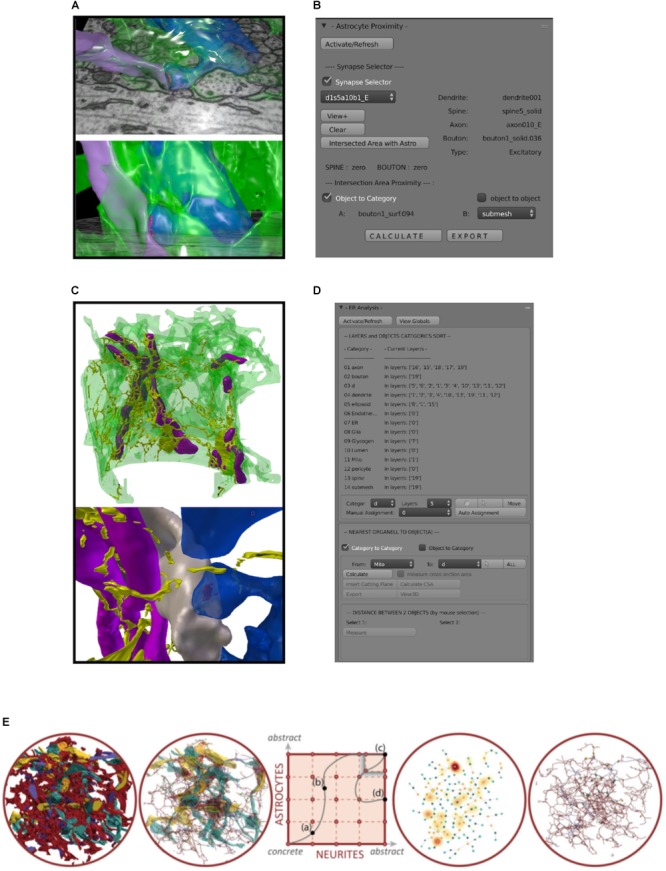
Custom made analysis tools. **(A)** Example of a 3D reconstructed astrocytic process (green, semi-transparent) enwrapping an axospinous synapse. Presynaptic terminal in purple, postsynaptic terminal blue. **(B)** Astroproximity Blender addon evaluates the contact surface area between astrocytic processes and synapses. **(C)** Example of a 3D reconstructed astrocytic process (green, semi-transparent) with its mitochondria (purple) and ER (yellow). **(D)** ER/mito Blender analysis addon measures minimum distance between ER, mitochondria and their closest synapse, as well as cross sectional area of the organelle. **(E)** Schematics of Abstractocyte, standalone software for qualitative observations of dense reconstructions of neuropil, specifically designed to analyze three-dimensional relationships between astrocytes and neurites. From left to right, a very occluded view including reconstructed 3D models of astrocytes and neurites can be simplified by showing astrocytes as their skeleton (second panel), to a full abstract view of connecting dots representing contact points between astrocytes and boutons/spines, whose halo highlight glycogen absorption maps (fourth panel), or a hybrid abstract view of astrocytes and contact points with boutons spines from panel one.

The specific need to study brain metabolism was the major driving force to partially customize ConnectomeExplorer for queries specific to glycogen (and other subcellular organelles) in glial cells segmented from high resolution, synaptic detailed, densely reconstructed EM stacks. A strategy was designed upon a dataset from aging mice brain (Calì et al., 2018) to simplify the complex NGV to an abstract 2D visual paradigm. Requirements specific to the analysis of the NGV lead to a standalone application for visual comparisons of astrocytic, neural and vascular processes at various levels of abstractions called Abstractocyte (**Figure 6E**; Mohammed et al., 2017). The application contains a 2D abstraction space (**Figure 6E**, center-squared panel) for visualizing astrocytes and neurons together at a particular degree of abstraction that can be chosen independently for each of the two categories and followed as a point in the abstraction space. Interactively moving this point allows them to smoothly transition between different abstraction levels in an intuitive manner (**Figure 6E**, left to right: different levels of abstraction from the same objects). In contrast to simply switching between different visualizations, this preserves the visual context and correlations throughout the transition. Users can smoothly navigate from concrete, highly detailed 3D views to simplified and abstracted 2D views.

#### Quantification of Cellular Structures

The increasing availability of high-resolution reconstruction of 3D models of cells leads to the development of specific tools for high accuracy quantification and interactive measurements in a way that enables scientists to discover peculiarities of cellular structures and to be able to perform fast and reliable statistical computations. In this phase of the investigation, particular efforts were taken to address these emerging trends and provide state-of-the-art solutions fulfilling and, if possible, anticipating the explicit needs of neuroscientists. Specifically, the main goal of this phase was to study and develop novel methods for extracting biological information based on intracellular organelle morphologies (e.g., endoplasmatic recticulum, mitochondria), distribution patterns (e.g., glycogen localizations) and functions (e.g., synaptic activity).

**FIGURE 7:**
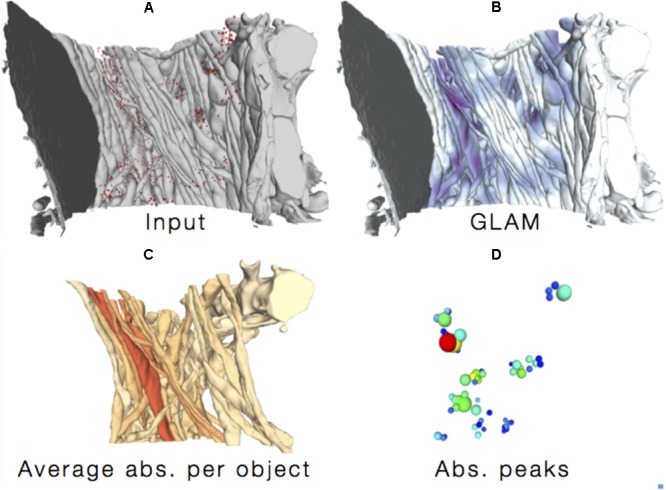
Glycogen Lactate Absorption Modeling (GLAM). Using as input **(A)** the accurate 3D reconstructions of cellular processes and the distribution of glycogen granules, a radiance-based model **(B)** estimates lactate absorption in order to highlight specific absorption patterns **(C,D)**.

The first main focus was to understand and describe to what extent astrocytes interact with the various elements of the neuropil (e.g., axons, dendrites, boutons, spines, synapses, blood vessels; **Figures 6, 7**). To this end, the capabilities provided by Blender, powerful and customizable open-source modeling and rendering software, were exploited to develop custom plug-ins for real-time interactive exploration and quantitative analysis, using python and Matlab scripts. In general, modules like Neuromorph^3^ (Jorstad et al., 2015) offer the possibility to analyze basic features of 3D models directly (e.g., lengths, surface areas, volumes). This possibility represents a substantial step forward in neuroanatomy compared to the standard analytical approach based on statistical analysis on many 2D images. Our solution involves the use of Blender integrated with the Neuromorph package, where we perform 3D analysis on a customized viewport of the software.

The morphological arrangement between astrocytic processes and synapses is one natural question that can be easily addressed using 3D models. Such analyses have been attempted in the past on sparse reconstructions (Ventura and Harris, 1999; Genoud et al., 2006; Ostroff et al., 2014), but we aimed to perform similar measurements in a more automated, therefore unbiased, way in order to screen larger, densely reconstructed volumes. A collection of tools for characterizing glia according to morphology and skeleton representations are currently being developed as add-ons on top of Blender software, a Python API or as a stand-alone component (iGraph python), for computing the area of all contact surfaces between boutons, spines and their surrounding astrocytic processes (**Figures 6A,B**; astroproximity).

Endoplasmatic reticulum (ER) and mitochondria are two structures of interest that have been reconstructed inside astrocytic processes, whose distribution might have an impact on phenomena such as calcium oscillations and energy balance (Magistretti and Allaman, 2018; Savtchouk and Volterra, 2018). Useful quantitative information to be extracted included the distance between these organelles and their closest synapse (as synaptic activity supposedly triggers their activation), and the cross-sectional area and perimeter of the objects at those sites. To extract these numbers, we coded a custom tool specifically designed to extract these features. As it is based on a nearest neighbor search method, the user interface was designed to allow users to use the same strategy for similar analysis (**Figures 6C,D**).

Finally, we designed an improved glycogen distribution analysis paradigm by highlighting possible sites for lactate uptake from neighboring neurites (Calì et al., 2017; Agus et al., 2018a), without the bias of considering only synapses (Calì et al., 2016) as sites for the ANLS (Pellerin and Magistretti, 2012; Mächler et al., 2016). The method is called *Glycogen-derived Lactate Absorption Map* (GLAM) and takes as input the high-resolution reconstruction of neural structures (**Figure 7**, top left), as well as a list of energy sources in the form of glycogen granules and computes an influence map according to a radiance transfer mechanism by considering a photon mapping analogy (i.e., individual glycogen granules are treated as sources of light; **Figure 7**, top right). Areas of greater glycogen concentration highlight portions of the cellular plasma membranes where the glycogen-derived lactate shuttling is more likely to occur (**Figure 7**).

A GLAM map can be easily integrated either in the Blender toolset for quantitative analysis or in ConnectomeExplorer; also, we processed it to extend to immersive VR (presented more in detail in WF4 section) equipment for providing both collaborative, fully immersive, stereoscopic exploration and analysis of the 3D datasets with unprecedented precision and resolution (Calì et al., 2017; Agus et al., 2018a). The system was tested and successfully used to carry-out the analysis of the intracellular spatial distribution of granules of glycogen.

### WF3 – Biochemical and Physiological Modeling

Biophysical modeling has a long tradition in neuroscience since the pioneering work of Hodgkin and Huxley (1952). Their framework for modeling the ion channels that give rise to action potentials is so powerful that it has allowed description of all subsequent ion channels, almost in a plug-and-play fashion (Harris J.J. et al., 2015). The situation for other biological modelers, however, is less straightforward. Modeling of subcellular metabolic processes, such as energy metabolism for example, is as not favorable. Due to experimental limitations, it is not yet possible to investigate these mechanisms in the laboratory in as great detail as electrophysiological techniques allow us to probe transmembrane currents, plus the number of interdependent reactions is larger. In addition, most experimental data available at the moment suffer from relatively poor spatial and temporal resolutions. These limitations can lead to any number of issues in designing models of brain energy metabolism (Jolivet et al., 2010). As a consequence, one needs to remain realistic about the level of details that is achievable in designing such models and second, these models need to be thoroughly constrained with the most informative datasets. This is the approach that we took in our original NGV model that, while being multi-scale within a few compartments, lacked biologically realistic ultrastructure (Jolivet et al., 2015).

#### Evolution of the NGV Model

In the early 2000s, Aubert and Costalat started developing models of various complexities addressing the compartmentalization of brain energy metabolism between neurons, glial cells and the vasculature (Aubert et al., 2001, 2002, 2005; Aubert and Costalat, 2002, 2005, 2007; Valabrègue et al., 2003). Their models drew heavily from models that had been designed based on the metabolism of erythrocytes (Heinrich, 1996). Their approach proved successful in qualitatively reproducing some hallmark experimental results, but it fell short of providing a flexible framework for modeling brain energy metabolism.

**FIGURE 8:**
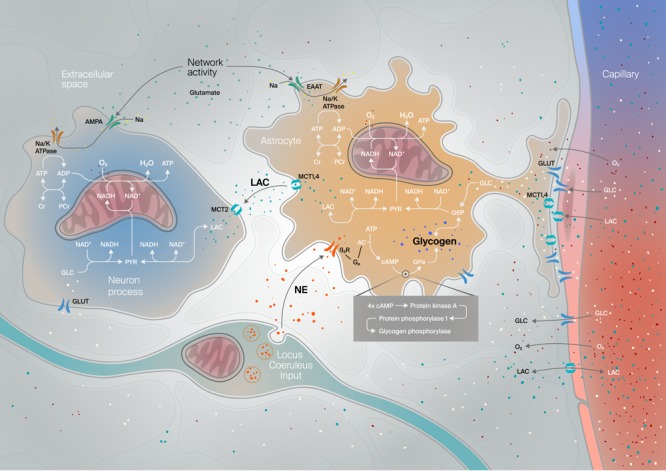
Noradrenergic modulation of energy supply in the NGV. Schematic compartmental diagram of the NGV model with noradrenergic (NE) locus coeruleus inputs along with neuronal, glial (astrocyte), vascular and extracellular space compartments. Key metabolic and transduction pathways for neuromodulation, glucose metabolism, energy production (ATP), glycogenolysis and lactate (LAC) shuttling from the astrocyte to the neuron are approximated. GLC, glucose; NAD(H) nicotinamide adenine dinucleotide (reduced); G6P, glucose-6-phosphate; PYR, pyruvate, EAAT, excitatory amino acid transporter (for glutamate); Na/K ATPase, ATP-dependent sodium-potassium pump; MCT, monocarboxylate transporter (for LAC); (P)Cr, (phospho)creatinine; GLUT, glucose transporter; AMPA, alpha-amino-3-Hydroxy-5-methyl-4-isoxazole propionic acid - type glutamate ionotropic receptor; O_2_, molecular oxygen; H_2_O, water; G_p_, G-protein; β2R, beta2-adrenergic receptor; AC, adenylate cyclase.

Building on their work, we developed a new model with a number of significant improvements (**Figure 8**; Jolivet et al., 2015). These are: (i) explicitly modeling the compartmentalization of nicotinamide adenine dinucleotide (NADH) between cytosolic and mitochondrial compartments; (ii) explicitly modeling glutamate as the signal driving electric and metabolic activities; (iii) establishing a link to the Hodgkin-Huxley formalism; (iv) explicitly and continuously updating reversal potentials based on intracellular concentrations (these are normally taken as constants in the Hodgkin-Huxley formalism even though the experimental literature suggests that they can vary very significantly during neuronal activity, see references in (Jolivet et al., 2015); and finally (v) constraining the model on what we judged was the most informative experimental dataset available at the time (Kasischke et al., 2004), as it offered a unique window into the subcellular and cellular compartmentalization of energy metabolism. In order to perform this last step, we devised a method inspired by flux-balance analysis to reduce the number of free parameters over which we would calibrate the model (see Jolivet et al., 2015 for details), and then optimized those parameters with the goal of reproducing the NADH transients that Kasischke and colleagues had observed in their experiments.

#### Development, Design and Discovery

We continued the development of the original NGV model to help guide the design of experimental approaches and as a tool to discover new NGV properties that would be inaccessible to laboratory techniques. The true measure of success for any modeling approach is the comparison of its predictions to new experimental measurements. We thus proceeded to compare the predictions of our model against various *in vivo* datasets (the calibration dataset from Kasischke et al., 2004, was obtained exclusively *in vitro* in acute brain slices). As illustrated in our previous computational study (Jolivet et al., 2015), our model performs remarkably well when compared to *in vivo* data. Remarkably, this is true for both rodent and human data. Our model not only accurately predicts lactate and oxygen transients observed in rodents upon physiological stimulation (Hu and Wilson, 1997), but also accurately predicts lactate transients, the relative increase of glucose and oxygen utilization, the drop of the ratio of oxygen to glucose utilization and the blood-oxygen-level dependent (BOLD) signal observed in humans in similar conditions. More recently, we incorporated ultrastructural localization data from WF1 for glycogen granules in astrocytes into an expanded NGV model that reveals another dimension of lactate signaling (manuscript in review). In general, we are at the stage in the development of our process to introduce more biologically realistic ultrastructure into our growing model.

We have thus advanced the field to a stage where energy metabolism can be integrated with Hodgkin-Huxley formalism, including in complex multi-compartment models of neurons, and eventually of astrocytes when these become more readily available. We should, however, also note some limitations of our approach. Our model doesn’t explicitly model every step or every pathway important in brain energy metabolism. It takes a number of shortcuts, for instance in modeling the mitochondrial Krebs cycle and oxidative metabolism. Similarly, our model doesn’t explicitly model key elements of brain energy metabolism such as glycogen, the pentose-phosphate pathway or the pathways known to regulate neurovascular coupling. In Jolivet et al. (2015), changes in CBF are modeled as an additional external input to the model. There is thus room for additions and improvements. However, as already noted above, we are not at a level of description of the key processes for brain energy metabolism that allows plug-and play additions or subtractions of mechanisms as is possible, to an extent, for the Hodgkin-Huxley framework. Moving forward, it will thus be necessary to recalibrate future more extensive models on the most informative experimental datasets available at these future time points. Alternatively, it might be necessary to devise a different and more scalable approach for the design and calibration of such models. Importantly, models of metabolism will need to become scalable in space like multi-compartment neuron models are, and addition of realistic biological morphology is both another key challenge for the future as well as a means of solving existing scaling problems by providing critical spatial dimensionality to the biochemical reactions.

**FIGURE 9:**
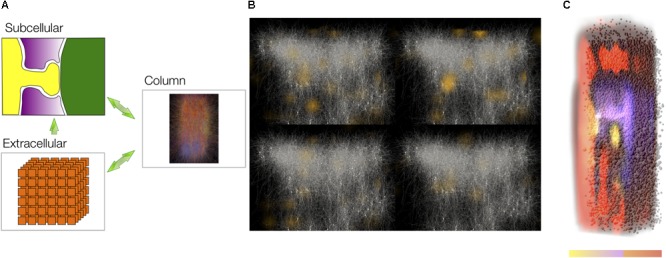
Metabolic simulation on the cortical column. **(A)** Voxelized space containing neurons and astrocytes was mapped onto cubic voxels overlapping the cortical column simulation. More depolarized neuronal processes are shown in red, while hyperpolarized processes are shown in blue. **(B)** Glutamate release at 10 ms intervals. Glutamate release drives the metabolic simulations. Neurons shown in white, glia in yellow. **(C)** The concentration of metabolites such as ATP (normalized) was saved as a time series and exhibits location-dependent effects.

The NGV model can be applied to functions beyond glucose processing. For example, we have used it to explore the involvement of glia in glutamate sequestration and recycling and the relationship of this function in energy consumption (Ahmed et al., 1990; Pellerin and Magistretti, 1994; Anderson and Swanson, 2000; Walls et al., 2009; Azarias et al., 2011). We mapped a large-scale simulation of spiking neurons onto cubic voxels 50 μm on a side (**Figure 9A**). Each voxel contained the set of differential equations describing metabolism. The glutamate concentration in each voxel, which instigates metabolic changes, was driven by synaptic events in the corresponding region of space in a largescale cortical simulation (**Figure 9B**). The concentration of metabolites could be visualized by subsequent analysis of the time series (**Figure 9C**).

The computational resources available for these simulations are provided by EPFL’s Blue Brain Project. While some limited scope simulations can be run on a single chip in a desktop or laptop computer, the more complex models require a multi-node supercomputer. The current machine employed for these simulations is called the Blue Brain 5 (BB5) and is located at the Swiss National Supercomputing Center (CSCS)^4^, in Lugano, Switzerland.

#### Clinical Relevance

The ultimate power of a biological model, beyond its academic or didactic utility, is its practical impact on solving real-world problems such as curing pathophysiological conditions. Among the most important medical applications of modeling will be assisting in unraveling the etiology of neurodegenerative diseases (Finsterwald et al., 2015) and then to aid in rational, guided design of treatments. Some of the disease states whose solutions are already being advanced by NGV modeling include those related to deficiencies in energy metabolism, specifically lactate or glycogen utilization. These can present clinically in a variety of forms including as mood disorders, sleep disturbances, addiction traits or psychiatric afflictions in various spectrums (Auer, 2004; Berthet et al., 2012; Bouzat et al., 2013, 2014; Cotrina et al., 2014; Petit and Magistretti, 2015; Boury-Jamot et al., 2016a,b).

### WF4 – Visualization and *in silico* Imaging

We designed and deployed a set of world-class software tools and hardware infrastructure to visualize and analyze the data reconstructed from microscopy stacks or created *in silico*. These tools were developed to enable simulation, visualization and analysis to be performed *in situ*, or on the same machine interactively, laying the foundation for exploiting the increasing power of supercomputers in simulation-based neuroscience in novel ways.

For prototyping, we have extended various open-source scientific visualization frameworks to explore static NGV datasets and analyze their structural aspects. These frameworks are limited in terms of capabilities and performance, as well. Consequently, we had to build a set of visualization tools capable of loading large datasets in the range of terabytes (TB) using out-of-core rendering methods. Moreover, we had to modify our in-house software tools, making it possible to combine the visualization of electrophysiological simulations of cyto-scale neuronal networks with glial cells. We also designed novel *in silico imaging* tools capable of visualizing the tissue models similar to how we see real brain tissue under the microscope, by simulating how the light interacts with the tissue in optical microscopes.

#### Livre

**FIGURE 10:**
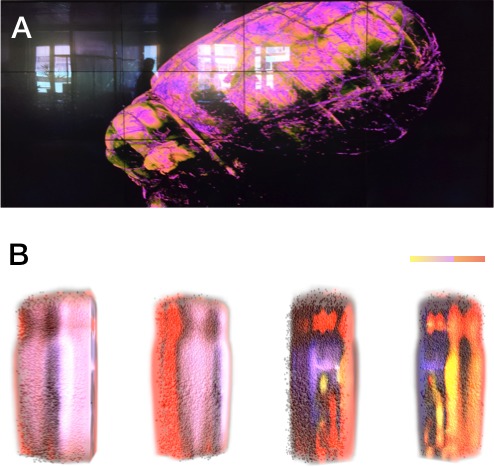
Visualization of Volumetric Data using Livre. **(A)** Visualizing the vasculature of a full brain dataset on a (3 × 4) tiled display wall. The visualization uses Livre to render the dataset out-of-core and the frames are streamed to the display wall using Tide. **(B)** Volume rendering of the ATP concentration change (normalized) in a neocortical column at 10 ms snapshots.

The microscopy stacks used in this project are quite large, in the range of tens of GB and can reach few hundreds of GB, which does not allow us to visualize them using standard software applications such as Paraview (Ayachit, 2015) or ImageJ (Collins, 2007). To overcome their limitations, we have developed an interactive volume rendering engine, called *Livre*, capable of handling datasets that are much larger than the main memory (Bilgili et al., 2013). Livre is an out-of-core, multi-node, multi-GPU and OpenGL-based volume rendering engine designed to load, visualize and analyze large-scale volumetric datasets. This tool is used for two types of applications: (1) visualizing volumetric stacks reconstructed from optical microscopes and (2) visualizing field data that can be voxelized from point cloud simulation. Examples for these applications of Livre are shown in **Figure 10**. **Figure 10A** shows interactive volume rendering of the vasculature of a full brain dataset reconstructed using the light sheet microscope. The size of this dataset is approximately 200 gigabytes (GB). **Figure 10B** shows a volume rendering of ATP concentrations in a neocortical column at 10 ms time snapshots.

#### Open-Source Visualization Tools

**FIGURE 11:**
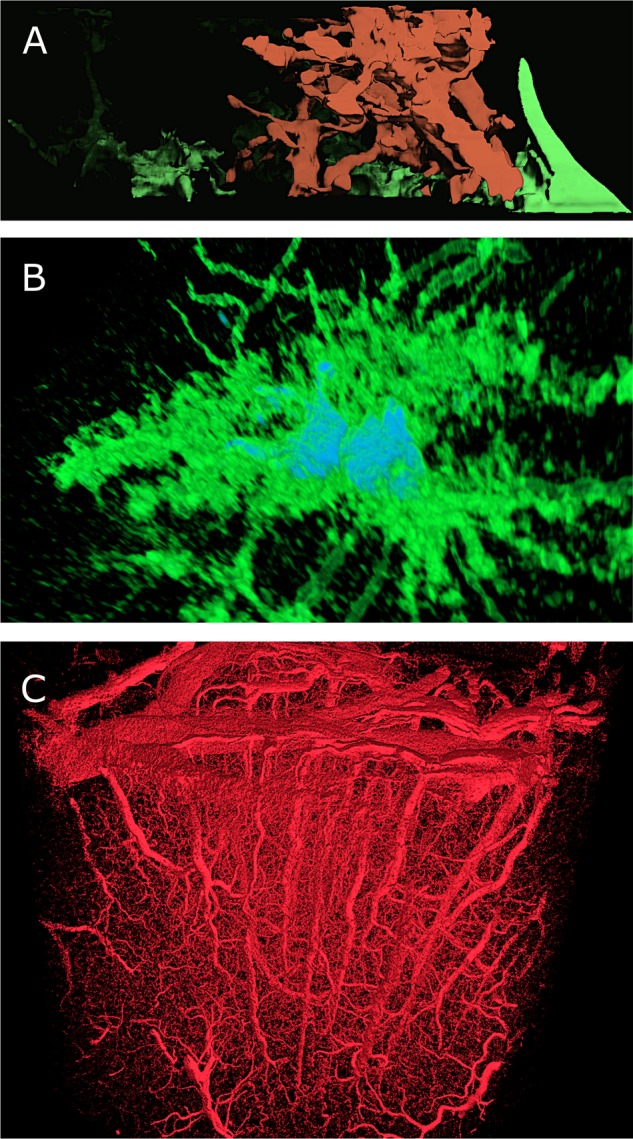
Scientific data visualization using open-source tools: ParaView and Voreen. **(A)** Volume rendering of a volumetric stack, containing an axon (red) surrounded by a glial cell, in green, using the volume rendering plug-in in ParaView. **(B)** Volume rendering of a light microscopic stack data, showing two astrocytes (in green) and their nuclei (in Blue) using ParaView. **(C)** Large volume stack of vasculature using the GPU-based volume rendering plug-in in Voreen. Dataset, courtesy of Bruno Weber et al., Institute of Pharmacology and Toxicology –Experimental Imaging and Neuroenergetics, University of Zürich.

During the initial phase of the project, we took advantage of various frameworks, based on open source software including Paraview (Ayachit, 2015), Blender (Kent, 2015) and Voreen (Meyer-Spradow et al., 2009), to visualize static glial cells and vasculature datasets. These tools were extended for prototyping reasons, mainly to validate the structural aspects of these datasets following their reconstruction. Paraview (**Figures 11A,B**) and Voreen (**Figure 11C**) are dedicated to create visual analytics of volumetric data using their volume rendering plugins. **Figure 11A** shows a reconstruction from a FIBSEM stack of an 8-weeks-old mouse somatosensory cortex. The glial cell, labeled in green, surrounds the dendrite of a neuron, colored in red. **Figure 11B** shows two apparently connected glial cells reconstructed confocal microscopy image stack. Voreen was useful for visualization the morphological skeleton of a vasculature dataset obtained with X-ray tomographic microscopy from the somatosensory cortex of a rat (Courtesy of Bruno Weber, University of Zurich, Switzerland). A rendering of this dataset if shown in **Figure 11C**.

**FIGURE 12:**
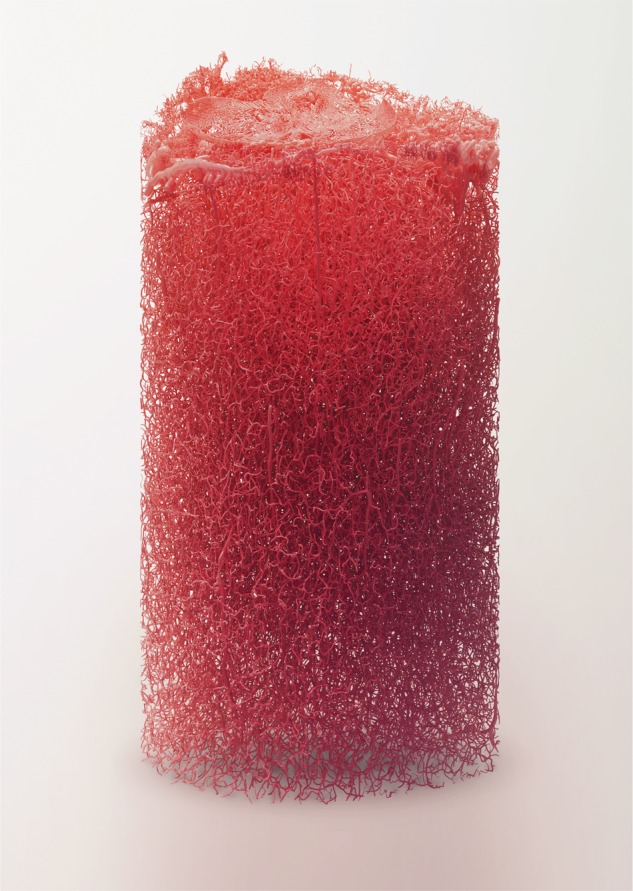
Scientific data visualization using open-source tools. Reconstructing a complex vasculature dataset using Blender-based Metaball algorithm and rendering the reconstructed model using Cycles, a physically plausible engine integrated in Blender.

Blender, a powerful rendering engine for reconstructing polygonal surface models from morphological skeletons, allowed creation of highly artistic rendering of various NGV datasets (**Figure 12**). The vasculature data is available as a skeleton that has only connectivity information represented by a set of connected points with specific radii. We implemented in Blender a convenient algorithm to reconstruct a polygonal mesh model of the vasculature using this skeleton. Then, we applied a highly realistic rendering algorithm to visualize this vasculature mesh using Cycles as shown in **Figure 12**. Moreover, Blender is used to create high quality visualizations of the NGV meshes that are directly reconstructed from EM stacks (dataset from **Figure 1**, rendered in **Figure 2**).

#### *In silico* Imaging

*In silico* imaging is defined as *the computer simulation of the entire pipeline of an imaging system including its source and detection components in addition to modeling the object being observed, reflecting its structural and functional aspects at different levels of detail* (Badano, 2011; Abdellah et al., 2017a). The type of simulation is, in general, used for developing novel imaging technologies and assessing their performance, to complement bench testing. The concept is adapted and applied in the context of simulation-based neuroscience, allowing us to visualize the NGV models as if they are seen under a microscope in the lab.

This approach requires building digital reconstructions of the tissue models at different scales, taking into account their interaction with the imaging system. For example, simulating tissue imaging with optical microscopy entails integrating the optical properties of brain structures into the tissue model to be capable of simulating the light interaction with the tissue. In the context of this workflow stage, a novel physically plausible method was designed and applied to visualize the tissue models by simulating the imaging pipelines of three different optical microscopes including brightfield, epi-fluorescence and light sheet fluorescence microscopy (Abdellah et al., 2017a,b).

**FIGURE 13:**
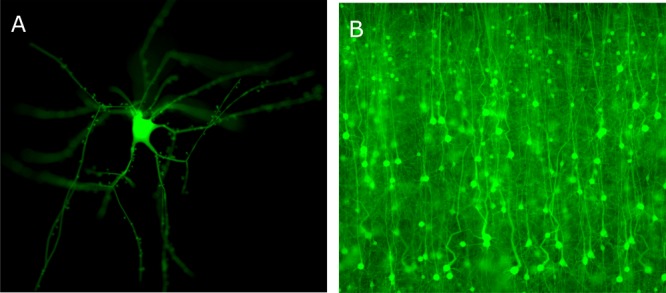
*In silico* physically plausible rendering. **(A)** Physically plausible *in silico* epi-widefield fluorescence imaging of a single neuron virtually tagged with GFP. The focal plane of the microscope is focused on the soma. **(B)** Physically plausible *in silico* epi-widefield fluorescence imaging of a digital slice reconstructed from the somatosensory cortex of a P14 rat.

**Figure 13A** shows an *in silico* fluorescence image of a single neocortical neuron tagged virtually with green fluorescent protein (GFP), and **Figure 13B** shows imaging a large scale digital slice of few thousands of cells at a specific focal distance. The tissue reconstruction (Abdellah et al., 2017b) is implemented in three steps: creating piecewise watertight mesh models from the morphological models of the neurons, creating volumetric models from the mesh ones, and finally the volumes are tagged with the optical properties of the brain at the somatosensory cortical region and also with the fluorescent proteins that are expressed in the tissue.

#### Virtual Reality

##### CAVE (Cave Automatic Virtual Environment)

**FIGURE 14:**
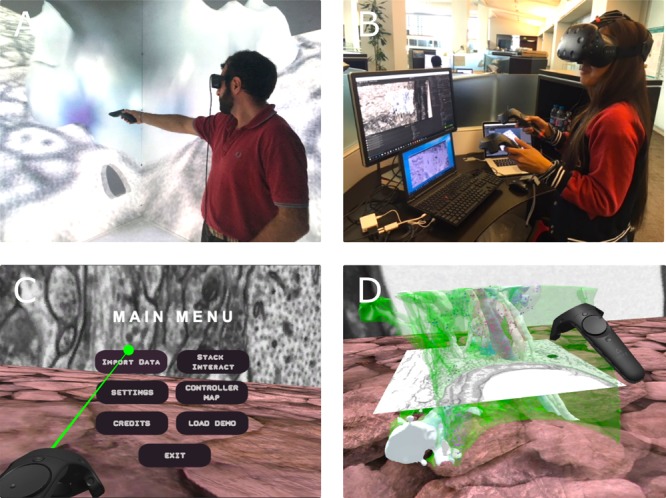
Use of VR to validate the Glycogen Lactate Absorption Modeling (GLAM). **(A)** GLAM map is used for planning sparse reconstructions in collaborative sessions performed on large-scale VR setups (CAVE), **(B)** and for intensive visual analysis on HMD-based stereo setups. **(C)** VR GUI implemented in unity to interact with the 3D models in virtual reality. **(D)** Loaded model in VR as seen during interactive navigation using HTC VIVE. Written consent from individuals appearing in this figure have been obtained for publication.

The CAVE system consists of 6 rear-projected screens, each with a ∼15-megapixel resolution (**Figure 14A**). The system stereoscopically projects images into a pair of 3D glasses, where resolution adds up to a total of ∼90-megapixel per eye. The six screens all together form a 3 m-by-3 m-by-3 m cubic room of four walls, plus the ceiling and the floor. The main end user (appointed as the person leading the visualization session) must wear a special set of glasses that are designed to work with the system. The glasses along with specialized tracking and rendering PCs, work together with the CAVE system to track the end-user’s head position and sense his/her movement. Consequently, all information is synchronized with the projectors including reporting user’s gaze. This allows the rendering PC to perform rendering calculations relative to the user’s eye gaze and drive the projectors accordingly. The result in the end is stable scenery of consistent stereoscopic images viewed through the end users glasses (DeFanti et al., 2011; Coburn et al., 2017).

Cave automatic virtual environment is considered an invaluable asset for scientific data visualization. In Calì et al. (2016) the system was utilized to perform explorative analysis sessions. Given the full immersivity of CAVE, scientists were able to collaboratively step inside a 3D world where cellular reconstructions are visualized and scaled out to be 2 million-fold larger (μ to m). This collaborative session eventually led to the observation of a non-random distribution of astrocytic glycogen granules. Thus, it laid the foundation for a development framework of analysis tools to further explore these findings. Our workflow for visualizing complex 3D models in CAVE involves Blender, an open source customizable 3D modeling software along with NeuroMorph add-ons (Jorstad et al., 2015), both are set to be the source view window where CAVE 3D models are loaded. This process is complemented by third-party software to handle translations between the two views. This operation is done via commercially licensed software called TechViz^5^ (Calì et al., 2016) (**Figure 14A**).

An alternate tool set for visualizing 3D reconstructions in CAVE is Unity, a game engine created by Unity Technologies mainly for developing 2-3D video games, and Mechdyne’s Get Real 3D, a Unity plug-in. In this setting, Unity plays the same role as Blender in modeling complex cellular structures to be visualized in CAVE, while Get Real 3D handles translating the complex structures created in Unity into the VR environment within CAVE.

##### HMD (Head Mounted Display)

An HMD is a head-wearable device designed with 2 display panels placed at close proximity to the user’s eyes (**Figure 14B**). It includes special optics located between the screen and eye in order to enhance focus back on the screen. Each of the two display panels presents separate images to each eye leading to having the user perceive them as 3D. In addition, the two displays track the orientation of the entire device, hence, the user’s head. This tracking system corresponds with the VR camera and is treated as input when developing VR applications (Coburn et al., 2017). Consequently, the user can look around and explore 3D reconstruction data in a high immersive VR environment.

Head mounted displays are considered consumer grade VR hardware or low-cost VR technologies as opposed to CAVE, which is still considered a large capital investment for most institutions. Over the past couple of years HMDs demonstrated technological advancements concerning resolution, field of view (FOV) and tracking, allowing them to rival the capabilities of CAVE (**Figures 14A,B**, respectively). In addition, a number of HMD features were accepted as solutions to some of CAVE’s drawbacks, such as portability, cost and ease of setup. Assembling and disassembling an HMD’s system is effortless and requires only minimal physical space. Current research studies employing VR-HMDs are still ongoing. This includes scientific applications involving surgical training (Karvonen et al., 2017; Huber et al., 2018) and psychological interventions (Laver et al., 2015; Chirico et al., 2016).

The HTC Vive (**Figure 14B**) and the Oculus Rift CV1 HMDs are two excellent lightweight solutions for carrying visualization tasks and interactivity in 3D space, with the former being a few steps ahead when it comes to FOV and tracking area (Huber et al., 2018). Consequently, we exploited the HTC Vive for the development of a VR visualization and interactive analysis tool called VR Data Interact, deposited in a Dryad repository (**Figure 14C**; Agus et al., 2018a,b). We adopted the Unity engine (version 5.6.3) as our development environment where we used readily available assets made for Unity such as the Virtual Reality Tool Kit (VRTK) and SteamVR. Standard assets and third-party library scripts were often modified or complemented with new ones in order to suit the application requirements. With VR Data Interact, the end user can engage in an explorative analysis session involving proofreading and driving hypotheses by viewing correlations between the reconstructed cellular structure and its superimposed 2D EM slice. Data interaction is demonstrated in the selection of a target model while actively scrolling along its corresponding z-stack (**Figure 14D**).

From a software perspective, development work with the HTC Vive is relatively supported with the device’s compatibility with commonly available Software Development Kits (SDKs) and development environments, not to mention (from a hardware perspective) its ability to be driven by a standard PC. However, as data become more complex and large, the hardware architecture becomes more demanding concerning GPU and memory as well as storage.

One disadvantage with the HTC Vive, and HMDs in general, is the loss of multi-user communication; due to the device design that obscures the end user’s face making his or her view and interaction with the real world blocked. With that in mind, the HTC Vive is designed to yield a room scale VR experience that can be enhanced with the integration of a high-level API allowing multiuser networking sessions to take place. Instantiating visualization sessions on multiple machines across different physical locations can place HMD technology and CAVE on equal footing when it comes to multi-user interaction and collaboration.

## Summary

**FIGURE 15:**
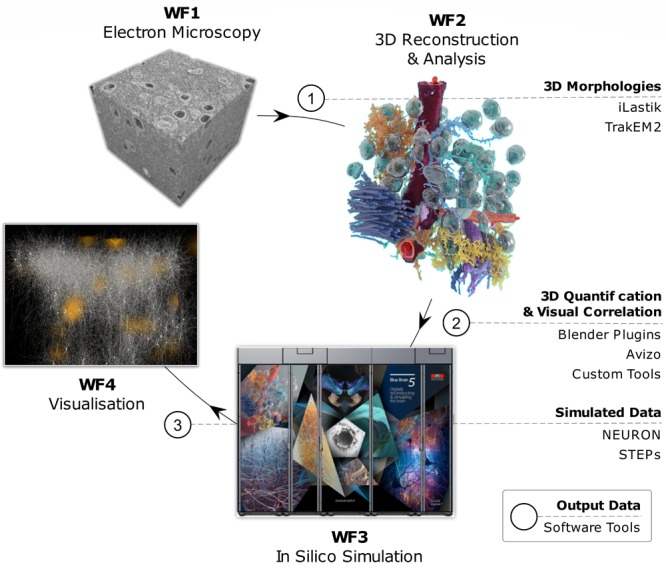
Infographics of the digitalization, modeling, simulation and visualization pipeline. WF1-WF4 are shown in sequence with example images from each step in the process, along with Output Data (bold lettering) and Software Tools corresponding to each WF step.

The quest for realistic and scientifically accurate representations of cytoscale biology requires a series of advanced methods. We describe in this report our current state-of-the-art process for achieving this goal, from probing of cell microstructures with EM techniques to reconstructions of cell volumes, models of biochemical pathways, and visualization tools including *in silico* imaging (**Figure 15**). Though we use brain energy metabolism in the NGV as a case in point, this workflow can be used for any cellular or oligocellular system. Our future plans call for progressively more detailed structural features with which to delimit ever more extended multi-scale and multi-compartment simulations.

## Author Contributions

JC and CC wrote the majority of the manuscript with contributions from other authors. CC prepared the tissues and performed the electron microscopy (WF1). CC and KK worked on the image segmentation, 3D reconstructions, and 3D analysis on the morphologies (WF2). MAg, DB, HL, and MH wrote the codes for the analysis and visualization tools (WF2). JC, DK, and RBJ contributed to the mathematical modeling and simulation of the NGV described in WF3. DB wrote the codes for the Virtual Reality Visualization and Analysis (WF4). MA, SE, and FS contributed the *in silico* imaging section (WF4). CC and PM coordinated the work from WF’s 1 and 2. HM, FS, DK, and JC coordinated the work from WF3 and 4.

## Conflict of Interest Statement

The authors declare that the research was conducted in the absence of any commercial or financial relationships that could be construed as a potential conflict of interest.

## Footnotes

1 http://openworm.org

2 ilastik.org

3 https://neuromorph.epfl.ch/

4 http://www.cscs.ch

5 http://www.TechViz.net
